# Ominous Mediastinal Mass Revealing Ectopic Thyroid

**DOI:** 10.1155/crip/5583511

**Published:** 2025-11-28

**Authors:** Cameron Summers, Gabriella Morey, Susana Ferra

**Affiliations:** ^1^Department of Pathology and Laboratory Medicine, HCA Florida Westside Hospital, Plantation, Florida, USA; ^2^Nova Southeastern University Dr. Kiran C. Patel College of Allopathic Medicine, Davie, Florida, USA

**Keywords:** differential diagnosis, ectopic thyroid, histological examination, incidental, mediastinal mass, tracheal compression

## Abstract

Mediastinal ectopic thyroid is a rare condition in which benign thyroid tissue manifests in an area other than its normal anatomical location. This report describes a 63-year-old female who was incidentally found via chest CT scan to have a right paratracheal soft tissue mass compressing and displacing the trachea to the left. She underwent an elective resection revealing unremarkable thyroid tissue. Subsequent neck ultrasound confirmed the presence of cervical thyroid supporting a diagnosis of ectopic thyroid tissue. The epidemiology, clinical manifestation, diagnosis, and management of mediastinal ectopic thyroids are discussed within this case report. Accurate diagnosis and management of ectopic thyroid relies on a combination of imaging studies and histological examination to confirm the presence of the thyroid tissue. Treatment for ectopic thyroid tissue depends on the nature of the symptoms and the potential risk of malignancy.

## 1. Introduction

The differential diagnosis of soft tissue masses of the anterior mediastinum includes masses originating from the thymus, lymphomas, germ cell tumors, and cervical goiters extending into the mediastinum. A rare cause is ectopic mediastinal thyroid without an attachment to the cervical thyroid [1]. Mediastinal thyroid tissue could be a true ectopic tissue or mediastinal goiter which has its attachment to the goitrous thyroid gland due to fibrosis. An ectopic thyroid gland is defined as thyroid tissue that is not located anterolaterally to the second to fourth tracheal cartilages. Ectopic thyroid can be lingual (at the base of the tongue), sublingual (below the tongue), prelaryngeal (in front of the larynx), or can be found at other rare sites including mediastinum [2].

A mediastinal ectopic thyroid should be considered in the differential diagnosis of all mediastinal masses. Surgical excision is recommended for both the diagnosis and treatment of this condition, because of its potential for malignancy and compression of other mediastinal structures [3].

This report describes an asymptomatic 63-year-old female with an incidentally found right paratracheal mass compressing and displacing the trachea to the left. A diagnosis of ectopic thyroid was rendered after histopathologic evaluation of the resected mass and sonographic confirmation of native cervical thyroid.

## 2. Case Report

An obese 63-year-old female with a history of hypertension, atrial fibrillation, myasthenia gravis, diabetes mellitus Type 2 and hyperlipidemia presented to the ED with syncope, cough, and diarrhea. A work-up CT chest scan showed large foci of ground glass opacities in the left lung and right lower lobe and a well-circumscribed soft tissue mass in the right paratracheal stripe measuring 7.7 × 6.6 × 5.4 cm. The mass was distinct from the right thyroid lobe. The patient was diagnosed with pneumonia, admitted to the hospital, and eventually discharged after successful treatment of the infectious process. She was instructed to follow-up as an outpatient with a local pulmonologist regarding the paratracheal mass.

**Figure 1:**
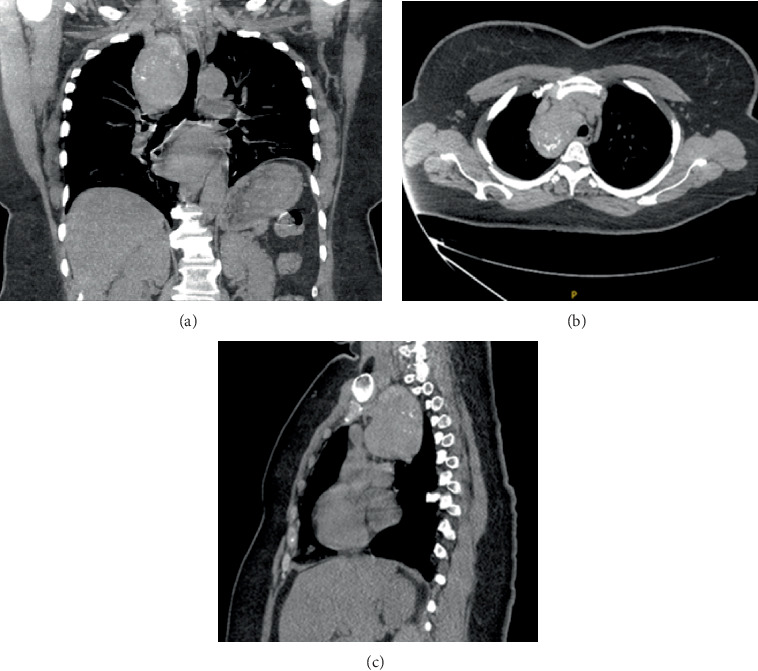
CT chest without contrast. Coronal (a), axial (b), and sagittal (c). Right paratracheal posterior mediastinum soft tissue mass 7.5 × 6.6 × 5.2 cm with few coarse calcifications and overall heterogenous density. The lesion displaces the trachea to the left.

She underwent a bronchoscopy with endobronchial ultrasound (EBUS)-guided FNA biopsy but the material was nondiagnostic. The patient was referred by her pulmonologist to a cardiothoracic surgeon. A repeat chest CT confirmed a right paratracheal soft tissue mass in the posterior mediastinum, measuring 7.5 × 6.6 × 5.2 cm. The mass contains a few coarse calcifications and displaces the trachea to the left, without compromising the airway lumen (Figure 1). There was no associated mediastinal lymphadenopathy. The thyroid was unremarkable on radiology. Additionally, the esophagus, heart, pleura, skeleton, and chest wall were reported with no significant findings. Imaging differential diagnoses included thymoma, lymphoma, and metastatic disease. Given the concern for neoplastic disease, she consented to surgical treatment and underwent elective video-assisted converted to open right thoracotomy with resection of the mediastinal mass and mediastinal lymph nodes.

**Figure 2:**
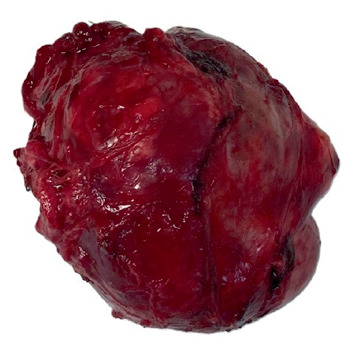
Gross photograph of the resected specimen. The lesion appears lobulated well-circumscribed, dark red and with a glistening surface.

**Figure 3:**
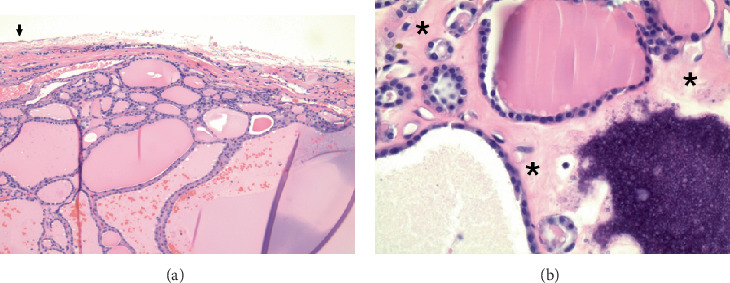
Photomicrographs of hematoxylin and eosin–stained sections. (a) 10x magnification demonstrating a well-circumscribed lesion with thin a capsule (arrow) and thyroid tissue with follicular architecture including different sized follicles, abundant colloid, and hemorrhage. (b) 40x magnification displaying areas of coarse dystrophic calcifications, interfollicular fibrosis (⁣^∗^), and a lack of nuclear atypia.

The resected mediastinal mass was submitted to pathology for intraoperative examination. The specimen consisted of lobulated dark red soft tissue with a glistening surface (Figure 2). It weighted 157 g and measured 8.5 × 7 × 6.0 cm. The cut surface revealed a multinodular architecture with colloid-like material and areas of fibrosis and calcifications. No hemorrhage or necrosis was present. Immediate interpretation rendered a diagnosis of tissue consistent with thyroid, no malignancy seen. Additional definitive histopathologic investigation confirmed the frozen section interpretation. The lesion was well circumscribed with a thin capsule and consisted of thyroid tissue in follicular arrangements of variable size with abundant colloid and hemorrhage (Figure 3a, H&E-stained section at 100 hpf). A high-power view revealed follicular cells with no nuclear morphologic abnormalities, fibrosis, and coarse calcifications (Figure 3b, H&E-stained section at 400 hpf). After confirmation of the presence of a cervical thyroid, the case was concluded as “benign ectopic mediastinal thyroid with multinodular hyperplasia.” The patient had an uneventful recovery and was discharged home after 3 days, with surgical care discontinued after a 1-month follow-up visit.

## 3. Discussion

Ectopic thyroid tissue refers to the rare condition in which thyroid tissue develops outside of its normal anatomical position in the anterior neck. Ectopic thyroid tissue can usually be found in areas along the path of descent of the thyroid gland, such as the base of the tongue or along the thyroglossal duct [4]. More rarely, it can occur in distant locations, such as the subdiaphragmatic breast, heart, larynx, and trachea. Mediastinal thyroid tissue, as seen in our patient, is an exceptionally rare entity with prior studies showing ectopic thyroid tissue in the mediastinum occurs in a very small percentage of patients with documented cases being sparse and often isolated [5].

Though the pathophysiology behind thyroid tissue migration to the mediastinum is unclear, it is believed that during embryonic development, abnormal descent, or developmental defects in the early stages of thyroid gland formation could cause the tissue to be displaced from its usual position [6].

Mediastinal thyroid tissue may present differently based on its location, size, and function. Many patients are asymptomatic, with the condition discovered incidentally during imaging or unrelated surgery. Symptoms can arise if the tissue is large or compresses nearby structures, causing chest pain, cough, or difficulty breathing. Functional ectopic thyroid tissue may lead to endocrine symptoms such as hyperthyroidism or hypothyroidism. Prior case reports highlight various presentations, from respiratory distress to incidental findings during routine exams [7].

Ectopic thyroid tissue can be managed conservatively if asymptomatic as there is low risk of malignancy. Medical management with levothyroxine supplementation can reduce the size of the ectopic tissue and thus any compressive symptoms [8]. One radiographic feature that is reported in literature is the fact that ectopic thyroid appears as hyperdense on CT and hyperintense on MR T1, this is thought to be due to the presence of thyroglobulin [9]. Although our case was treated through surgical management, asymptomatic cases are reported to be treated by regular follow-up as well [10]

When considering the diagnostic possibilities of a mediastinal mass, three entities represent the overwhelming majority of the lesions: lymphomas, germ cell tumors, and thymic epithelial lesions. According to the World Health Organization (WHO) tumor classification guide, nodular sclerosis Hodgkin lymphoma (NSCHL) makes up 20% of all mediastinal tumors and between 50% and 70% of mediastinal lymphomas. The patients present asymptomatically with a mass detected on an unrelated chest X-ray and some may report classic “B symptoms” which refers to the triad of fever, weight loss and night sweats. Histologically, characteristic Reed–Stenberg cells, the hallmark of NSCHL, stain positive for CD30 and CD15 with weak PAX5 expression. In our case, the lesion is extremely well circumscribed different from the infiltrative nature of NSCHL and no systemic symptoms were present making it an unlikely possibility. Seminoma, another entity that occur in the mediastinum, is almost entirely in post pubertal males. It accounts for 10%–37% of mediastinal germ cell tumors. Unlike NSCHL, the majority of these patients are symptomatic with patients primarily presenting with dyspnea, chest pain, and weight loss. Radiographic imaging will reveal a lobular homogenous mass. A comprehensive physical exam is important to rule of a metastasis from the testicles. On histology, these lesions appear as nests of polygonal cells with prominent nucleoli and clear cytoplasm separated by fibrous tissue, the background of these nests harbors a lymphoid infiltrate. The neoplastic cells typically stain for OCT4, SALL4, and CD117. Lastly, thymic epithelial tumors make up 25%–30% of the lesions in this anatomical region, majority of which are thymomas. The patient often develops autoimmune diseases such as myasthenia gravis, like is the case of our patient. Thymomas are diagnosed primarily on their morphology by which they are also subtyped. [11–13]. Given the multiple diagnostic possibilities, each with a potential different treatment, in the presence of a mediastinal mass, histology remains the standard to establish the diagnosis.

Accurate diagnosis and management rely on recognizing this uncommon presentation and using a combination of imaging studies and histological examination to confirm the presence of the ectopic thyroid tissue. Chest X-rays, CT scans, and MRIs may reveal an abnormal mass in the mediastinum, which should then prompt further investigation. Biopsy or surgical resection of the mediastinal mass is needed to provide definitive evidence of thyroid tissue as exclusion of other more common entities requires histological confirmation. Once thyroid tissue is identified, exclusion of thyroid malignancy becomes paramount. Thyroid cancer metastases should always be considered and excluded before accepting the diagnosis of ectopic thyroid. Both metastatic thyroid carcinoma and, more rarely, de novo thyroid malignancy within ectopic thyroid have been reported [14, 15]. Papillary thyroid carcinoma (PTC), the most common form of thyroid carcinoma, is straightforwardly excluded by the lack of architectural and cytomorphologic nuclear features characteristic of the entity. In the case of follicular carcinoma, histology by itself can be fairly similar to normal thyroid, making the distinction challenging. The presence of a dominant nodule with capsular or vascular invasion or prior history of thyroid surgery are clues to suspect malignancy. In our patient, histomorphology easily excluded PTC. The presence of peripheral coarse calcifications is suggestive of a slow growing process in keeping with a benign lesion. Additionally, there was no dominant nodule within the ectopic thyroid, there was no history of thyroid surgery or malignancy, and no nodule was present in the cervical thyroid supported by unremarkable findings in sonography and CT studies, all reassuring in the exclusion of carcinoma.

Treatment for ectopic thyroid tissue depends on the characteristics of symptoms and concerns about malignancy. If conservative management fails or is contraindicated, surgery may become the first-line treatment, with radioactive iodine or levothyroxine therapy as adjunct options [16].

## 4. Conclusion

Ectopic thyroid tissue is a rare condition in which thyroid tissue develops outside of its normal anatomical position in the anterior neck. It is important to consider ectopic thyroid tissue in the differential diagnoses of a mediastinal mass. They are often incidental findings on imaging. Accurate diagnosis and management rely on a combination of imaging studies and histological examination.

## Data Availability

The data that support the findings of this study are available from the corresponding author upon reasonable request.
